# Mental and Emotional Health of Children Exposed to News Media of Threats and Acts of Terrorism: The Cumulative and Pervasive Effects

**DOI:** 10.3389/fped.2016.00026

**Published:** 2016-03-23

**Authors:** Marie Leiner, Jesus Peinado, Maria Theresa Malazo Villanos, Isis Lopez, Ricardo Uribe, Indu Pathak

**Affiliations:** ^1^Pediatrics, Texas Tech University Health Sciences Center, El Paso, TX, USA; ^2^Paul L. Foster School of Medicine, Texas Tech University Health Sciences Center, El Paso, TX, USA

**Keywords:** violence exposure, pediatric populations, terrorism news, disparities, trauma

Viewing extreme violence and terrorism, either directly by witnessing acts or indirectly by watching them in the media, affects children’s mental and emotional health (1), and some children are at a higher risk for negative effects than others. Indirect exposure to terrorism acts and threats through the media affects the mental health of children, in both short- and long-term ways that differ completely from the effects in adults. Children’s vulnerability, immaturity, and developmental state change their perspective, and the tools used to confront these issues do not affect each child equally. Additionally, emotional problems might not surface immediately; instead, they can remain latent until they surface eventually. How and when this occurs depends mostly on additive effects generated by the environment in which the child develops and additional disparities in which the child confronts.

The Federal Bureau of Investigation (FBI) defines terrorism as “the unlawful use of force or violence against persons or property to intimidate or coerce a government, the civilian population, or any segment thereof, in furtherance of political or social objectives” (2). Direct or indirect exposure to terrorism violence provokes a state of terror in the general public through the use of calculated acts of violence, which includes murder, mutilation, and explosions that affect innocent people, often including children. Even though the events might no longer be in the news, the psychological scars and trauma caused by viewing the horrifying images and by the memories are pervasive and do not heal easily (3). The natural tendency of adults to try to forget about the problems and move on with their lives often results in underestimating the importance and effect of the exposure on the emotional and psychosocial problems of children.

The negative effect of exposure to violence in the media on children has been extensively discussed for years, with many controversial and conflicting results and conclusions. While some have found highly negative effects (4–6), others have denied any possible effects from media exposure (7, 8). Nevertheless, the existing evidence about the effects of media exposure to terrorism and violence acts among children should not be underestimated or dismissed. The point of discussion here is to consider the need to support strategies that reduce the effects of unsupervised viewing of terrorism acts and threats presented by the media. While stopping the media presentation of this violence might not be possible, there is much room to consider strategies that can reduce and help to better understand the effects of violence on the emotional health of children at home, in school, and in health sector. The media itself can also help by increasing the attention given to these strategies.

## Availability and Repetition of Unrestricted Media

Children today have virtually unrestricted access to worldwide media coverage of terrorism acts, which threatens their mental and physical health due to their crude nature. Terrorist acts have been documented throughout history, but in the last decades, media coverage has given unfair attention to these despicable acts of shocking violence.

Media access brings worldwide events very close to children, and often in a very crude way, through the news, Internet, or social media. It can involve intrusive, unedited images portraying extreme acts of violence that can be very intense (9, 10). Children do not have a sense of spatiality and rarely understand the concept that these events have occurred far from their current location. Instead, these almost live events can cause feelings of unsafety, hopelessness, and helplessness, which are often externalized by conduct problems.

The effects of indirect exposure to violence affect younger children more than older children (11), and research indicates that real exposure to war events in children below age 10 increases their risk of developing internalizing disorders in later life (12). This was also demonstrated by two studies that compared exposure to drug cartel terrorism (collective violence) among children on both sides of the US–Mexican border. These studies included children at different ages (1.5–5 and 6–16 years) and examined the cumulative effect of poverty and collective violence ­(terrorism) (11, 13).

In Mexico, news about mass murders of men, women, and children was broadcasted repeatedly for days and months by multiple outlets, including radio stations, newspapers, and television networks. The media presented the death of activists and bystanders with extreme crudeness, including covering many acts of terror, such as mutilations, bombings, kidnappings, tortured bodies, and decapitations.

When comparing the psychosocial and behavioral problems of children (6–16 years old) on each side of the border, the studies found that, on the Mexican side, the externalizing of problems from exposure to collective violence increased. The younger Mexican children had a threefold increase in emotional and behavioral problems when compared to their US counterparts who were not confronted by this indirect mass media victimization. Young Mexican children also had higher scores than their US counterparts for most emotional and behavioral scales, including mild/severe brain injury (14); single-suture craniosynostosis (15); parental history of cocaine, alcohol, tobacco, and marijuana exposure (16); prenatal cocaine exposure (17); maternal current and past depression (18); and hearing impairment (19). The excessive amount of exposure to viewing violent events can have a profound effect on children’s sense of security, leaving strong feelings of vulnerability and resulting in emotional and psychological trauma, even though these events are only presented by the media (20–22). These studies have some limitations, including their cross-sectional study design. In addition, given the amount of news in the city about generalized violence, for these studies, it was assumed that families were indirectly victimized when they could be both direct and indirect victims of violence. Available information did not include specifics about this additional effect on the families.

## The Effect of Inequality and Adversity and Their Relationship with Exposure to Violence

Not all children are fortunate enough to live with parents who can provide them with the human and social experiences that are essential for children to help them thrive and grow into healthy, productive adults. Children confronting inequalities due to poverty and low socioeconomic status must contend with increased exposure to violent events during their life span (23), which will have a profound effect in their mental and physical health. Often, children living with these inequalities are at a higher risk of confronting direct and indirect victimization, which may be just one event in a series of adverse events. The emotional outcome of a child will always reflect the cumulative load of these stressful events.

The CDC-Kaiser Adverse Childhood Experiences (ACE) study demonstrated the harmful, immediate, and long-lasting effects that adversity has on child development (19). The immediate cognitive effects reported included attention problems, learning disorders, and poor school performance. Children who experience adversity-related problems may suffer decades later from chronic disease and mental illness and may display aggressive and violent behaviors as adults. In addition, direct or indirect exposure to violence is recognized as an adverse childhood experience that can become a form of victimization and is considered a basic cause of morbidity and mortality in adults (24–26).

## Child Development and Chronic Stress

Excessive or prolonged activation of stress response systems in a child can have damaging effects on learning, behavior, and health across a person’s life span. Constantly viewing threatening images and acts of violence produce episodes of stress that increases a child’s heart rate and blood pressure as well as the production of stress hormones. Because of their age, children do not have the resources to manage this stress, which can disrupt the development of brain architecture and other organ systems with lifelong consequences. The elevated stress levels may last longer than in an adult, interrupting the natural process of development causing physical cognitive and emotional problems (27, 28).

## Conclusion

Reports of terrorism events indicate that, in 2015, there were at least 21 terrorist attacks around the world where 50 or more people were killed. Six of those attacks killed more people than those who were killed in the France attacks (29). The events that get the maximum attention are often the deadliest or those perpetuated with the maximum hate, blood, and gore. Both direct and indirect effects of terrorism violence affect adults and have strong implications for their physical and mental health (20). Adults are better equipped to deal with both physical and emotional effects, and therefore accounting for the effects of this violence on children requires a broad understanding of children’s vulnerabilities (30, 31). Children’s developmental stages limit their abilities to describe their symptoms, communicate their wants or needs, and seek assistance (32).

Exposure to terrorism violence through the media, videogames, movies, or news about disasters and/or individual incidents has different implications for the pediatric population, and multiple studies have shown a link to adverse mental health effects in children. These findings emphasize the need and importance for primary care providers to receive training (33) to better assess and refer children who need therapy to confront and deal with these emotional effects. The effects of exposure to terrorism violence on children might not be apparent for several days, months, or even years. The effects are dosed and triggered by existing psychological problems, adversity, and/or previous victimization.

Addressing the effects of terrorism violence acts involves much more than just taking care of the direct victim’s needs, it requires considering that the bystanders watching these acts in the media are highly vulnerable as well. Planning appropriate preventative measures, including restricting media use, parental education, and early detection of emotional problems, can help prevent the problems that we confronted after media exposure during the September 11th terrorism events. The long-term effects of media exposure to terrorism events are still largely unknown, and the current mental health system has been ineffective in identifying and dealing with the mental health needs of children that these acts of terrorism have created (34, 35).

Early intervention services to improve outcomes for children have a tremendous impact on children’s lives as well as the lives of their families and society as a whole. Treatment for those at a higher risk is a recognized challenge, especially among those from a lower socioeconomic status or minority backgrounds. Referrals, if necessary, for follow-up may not occur in many cases due to economic, cultural, health literacy, language proficiency, and educational disparities. Therefore, an additional counseling is required to inform parents and youth about the benefits of follow-up therapy.

Opportunities to help families mitigate the harmful effects of adversity, inequalities, and exposure to violence need to be considered and nurtured to help make a difference in the life of a child. While the direct effect that wars have on children is well understood, the indirect effects that unrestricted access to terrorism views have on children is not only less understood but also important to understand. Dismissal of the detrimental mental and emotional effects on children, despite the available evidence, will only add to the societal burden from the emotional stress and suffering. With better awareness and proper measures to monitor children’s access to violent acts, we can improve their quality of life and the future of our society.

## Author Contributions

ML made substantial contributions to conception and design of idea, participated in drafting the article, and approval of the version to be submitted. JP made substantial contributions to conception and design of idea, participated in drafting the article, and approval of the version to be submitted. MV made substantial contributions to conception and design of idea, participated in drafting the article, and approval of the version to be submitted. IP made substantial contributions to conception and design of idea, participated in drafting the article, and approval of the version to be submitted. IL and RU made substantial contributions to idea discussion, revised critically the article, and gave final approval of the version to be submitted.

## Conflict of Interest Statement

The authors declare that the research was conducted in the absence of any commercial or financial relationships that could be construed as a potential conflict of interest.
